# In vivo detoxification of aflatoxinB1 by magnetic carbon nanostructures prepared from bagasse

**DOI:** 10.1186/s12917-014-0255-y

**Published:** 2014-10-30

**Authors:** Farhat Ali Khan, Muhammad Zahoor

**Affiliations:** Department of Pharmacy, Sarhad University, Peshawar, Pakistan; Department of Chemistry, University of Malakand, PO Box: 18000, Chakdara Dir (Lower), KPK Pakistan

**Keywords:** Magnetic carbon nanostructures, Poultry birds, Alanine transferase, Alkaline phosphatase, Bagasse

## Abstract

**Background:**

Aflatoxins are serious hazard to poultry industry and human health. Broiler chickens fed on aflatoxin contaminated feed develop various abnormal signs and behavior including less attraction toward feed, abnormal faeces consistency, growth retardation, dirty and ruffled feather, abnormal organs size and weight and blood serum biochemistry. Therefore the study was aimed to detoxify aflatoxin B1 in poultry feed. In this study a novel adsorbent was prepared from bagasse, characterized in vitro and in vivo it was fed to different groups of poultry birds along with aflatoxin B1. The groups were given arbitrary names A, B, C, D, E and F. Group A was fed with normal decontaminated feed, group B was fed with aflatoxin contaminated (200 μg/kg feed) feed while the groups C, D, E and F were fed with aflatoxin contaminated (200 μg/kg feed) feed plus 0.2, 0.3, 0.4 and 0.5% adsorbent respectively. Clinical signs and behavior of the chicks; blood level of alanine transferase, alkaline phosphatase, serum albumen, serum total proteins and serum globulin; Mortality; Body and organ weights; Hemorrhages in organs etc. were monitored in order to study the efficacy of the adsorbent for binding of aflatoxin B1 in the gastrointestinal tract of chickens. Statistical approach was adopted to analyze the data.

**Results:**

It was found that adsorbent amount 0.3%/kg feed was highly effective to adsorb and detoxify aflatoxin B1 in gastrointestinal tract of broiler chickens and pass safely leaving no harmful effects. However the results of groups E and F fed on 0.4% and 0.5% respectively showed slight variation in tested parameters from group A.

**Conclusions:**

The prepared adsorbent was efficient for the detoxification of aflatoxin B1 in gastrointestinal tract of chicks and no negative symptoms associated with the use of activated carbon as previously reported were observed for the adsorbent under study.

## Background

Pakistan is situated in the subtropical region in the world globe. Its exact position lies between 24° and 40° North latitudes. As the climatic conditions of the subtropical regions are warm, moist and damp, therefore it provides best condition for development of fungus in poultry feed that is a threat to poultry industry which is an important part of livestock sector having 52.2% share in agro based economy of Pakistan that contribute 19% of total meat production in the country [1]. Aflatoxins are produced by different species of *Aspergillus*. The contamination of aflatoxin in the feed may cause aflatoxicoses in poultry birds that leads to lower their growth rate, weight loss and anorexia. The increased susceptibility to environmental and microbial stresses caused by aflatoxins ultimately increases the rate of mortality [2–4]. Among aflatoxins, aflatoxin B1 is of predominant importance as it has highest toxic potential due to teratogenicity, carcinogenicity and mutagenicity [2,3].

A practical approach for the detoxification aflatoxins in poultry feed is the use of adsorbent [5]. As adsorbents have the potential to bind aflatoxins and inhibit their absorption from gastrointestinal tract [6]. However large quantities and negative interaction of adsorbents with nutrients are causes of great concern [7–9]. The activated carbon due to its high surface area and cheap availability is used for the adsorption of organic and inorganic pollutants [10]. However dehydration and salt deficiencies are encountered when it is administered to poultry birds [7–9].

Magnetic adsorbents have recently gained considerable importance as they can be removed from media by the use of magnetic field after use that can then be regenerated. Thus it will not cause any adverse effects on environment. However, small surface area and less adsorption capacity rendered these materials to be used as adsorbents [11]. Moreover their preparation requires several steps and special chemical procedures. According to Kahani *et al*. [10] the conversion of biomass into magnetic carbon nanocomposites would provide an excellent and cheap adsorbent for the remediation environmental problems.

If activated carbon or any other adsorbent is fed to poultry birds, they could not be easily collected from feaces and will results in secondary problems to environment. On the other hand if adsorbent is magnetic, it will be easily collected from feaces through magnetic process and will not result in secondary problems to environment. Keeping in view the mentioned goals, in this study magnetic carbon nanocomposites were prepared from bagasse and fed along with aflatoxin B1 to poultry birds in different concentrations. Different physiological as well as clinical symptoms were monitored to evaluate the effectiveness of the adsorbent.

## Results and discussion

As already stated that the prepared adsorbent was characterized by surface area analyzer, SEM, XRD, FTIR, TG/DTA and EDX (our submitted results). The prepared adsorbent was tested for its attraction toward a bar magnet. The composite was attracted by magnet which shows that it was magnetic which was further confirmed by XRD analysis and FTIR spectroscopy. The diffraction peaks at 2θ of 29.7, 35.7, 44.9, 54.15, 57.55 and 62.5 represents the corresponding indices 220, 311, 400, 422, 511 and 440 planes of cubic unite cells which correspond to the magnetite structure. The presence of magnetite and maghemite peaks indicated that the composite was magnetic. The FTIR spectrum showed Fe-O stretching at 596.65 cm^−1^. The BET surface area was 97.07 m^2^/g with total pore volume of 1.71 cm^3^/g. The particle size of the prepared adsorbent was determined by Debye-Scherer’s equation were found to be in the range of 70-350 nm. The SEM images showed that the shape of the Fe_3_O_4_ appear somewhat cubical whereas the sizes of iron oxide carbon nanocomposites estimated were found in the range of 60 to 300 nm. TG/DTA analysis showed mass loss in the range of 30°C to 70°C with DTA endothermic peak, while further mass loss was observed in the range 250-720°C with DTA exothermic peak. EDX analysis showed the presence of Iron, Oxygen and Carbon while Calcium was observed as impurity. The adsorption parameters of the in vitro analysis are shown in Table 1. The in vivo results of the study are outlined as follow.

**Table 1:** **Adsorption parameters of aflatoxin B1 on carbon nanocomposites prepared bagasse**

**Adsorbent prepared from:**	**Langmuir isotherm**			**Freundlich isotherm**		
Bagasse	Q_0_(mgg^−1^)	b	R^2^	K	1/n	R^2^
	66.68	0.25	0.991	9.3	0.714	0.998

### Clinical signs and behavior

Weekly results of clinical signs and behaviors of broiler chicks in different groups are presented in Table 2. The results revealed that chicks of group A showed normal signs and behaviors. All chicks were found active and alert upon taping the cage throughout the experimental weeks. They rushed toward feed and water, while feaces were found formed and semisolid. Feathers were noted shiny and clean till the end of experiment. Chicks of group B exhibited abnormal signs and behaviors throughout the course of study. The intensity of abnormal signs and behaviors of chicks in group B were observed less at the end of 1^st^ week which turned to maximum with the passage of time. Chicks of group B showed less attraction toward feed, while water intake was increased. Depression was observed during 1^st^ week and recorded maximum at the end of 6^th^ week. Feaces were noted soft and watery whereas feathers were examined dull and dirty at the end of 6^th^ week. Chicks of group C were rushed toward feed and water and were noted alert upon taping the cage, while feaces consistency were recorded normal during 1^st^ week. Abnormal signs and behavior in chicks of group C was started from 2^nd^week and was noted maximum at the end of 6^th^ week; however found very low when compared to group B. Groups D, E and F chicks were found active and showed more attraction toward feed and water. Feaces were formed and semisolid, feathers were remained normal, clean and shiny throughout experimental period. Though chicks of groups D, E and F exhibited normal signs and behavior but group E results were found closed to that of group A.

**Table 2:** **Clinical signs and behavior of broiler chickens given different levels of adsorbent prepared from bagasse**

**Time period**	**Clinical signs and behavior**	**Range of score**	**Groups**					
			**A**	**Aflatoxin B1 (AfB1) 200 μg/kg feed**				
**Week (day)**				**B**	**C**	**D**	**E**	**F**
1(7)	Alertness	0-3	0	2	0	0	0	0
	Normal-Depressed							
	Attraction to feed	0-3	0	4	0	0	0	0
	Normal-Less interest							
	Attraction to water	0-3	0	10	0	0	0	0
	Normal-More/Less interest							
	Faeces consistency	0-3	0	3	0	0	0	0
	Normal-Watery							
	Feather	0-3	0	0	0	0	0	0
	Normal-Ruffled and Broken							
Cumulative Score			0	19	0	0	0	0
2(14)	Alertness	0-3	0	6	0	0	0	0
	Normal-Depressed							
	Attraction to feed	0-3	0	10	3	0	0	0
	Normal-Less interest							
	Attraction to water	0-3	0	24	2	0	0	0
	Normal-More/Less interest							
	Faeces consistency	0-3	0	14	3	0	0	0
	Normal-Watery							
	Feather	0-3	0	0	0	0	0	0
	Normal-Ruffled and Broken							
Cumulative Score			0	54	8	0	0	0
3(21)	Alertness	0-3	0	8	3	0	0	0
	Normal-Depressed							
	Attraction to feed	0-3	0	10	3	0	0	0
	Normal-Less interest							
	Attraction to water	0-3	0	30	2	0	0	0
	Normal-More/Less interest							
	Faeces consistency	0-3	0	25	5	0	0	0
	Normal-Watery							
	Feather	0-3	0	0	0	0	0	0
	Normal-Ruffled and Broken							
Cumulative Score			0	73	13	0	0	0
4(28)	Alertness	0-3	0	12	3	2	1	2
	Normal-Depressed							
	Attraction to feed	0-3	0	27	4	2	1	1
	Normal-Less interest							
	Attraction to water	0-3	0	48	6	2	0	0
	Normal-More/Less interest							
	Faeces consistency	0-3	0	37	5	0	0	0
	Normal-Watery							
	Feather	0-3	0	0	0	0	0	0
	Normal-Ruffled and Broken							
Cumulative Score			0	124	18	6	2	3
5(35)	Alertness	0-3	0	15	3	1	1	1
	Normal-Depressed							
	Attraction to feed	0-3	0	51	5	2	1	1
	Normal-Less interest							
	Attraction to water	0-3	0	79	8	2	2	2
	Normal-More/Less interest							
	Faeces consistency	0-3	0	56	9	0	0	2
	Normal-Watery							
	Feather	0-3	0	0	0	0	0	0
	Normal-Ruffled and Broken							
Cumulative score			0	201	27	5	3	6
6(42)	Alertness	0-3	0	18	5	3	1	3
	Normal-Depressed							
	Attraction to feed	0-3	0	67	8	4	2	4
	Normal-Less interest							
	Attraction to water	0-3	0	89	11	6	2	5
	Normal-More/Less interest							
	Faeces consistency	0-3	0	75	14	0	0	2
	Normal-Watery							
	Feather	0-3	0	16	5	0	0	2
	Normal-Ruffled and Broken							
Cumulative score			0	265	43	13	5	16
	Total cumulative score			736	109	24	10	25

A = Basal feed, B = Basal feed + Aflatoxin B1 (200 ppm), C = Basal feed + Aflatoxin B1 (200 ppm) +0.2% Adsorbent, D = Basal feed + Aflatoxin B1 (200 ppm) +0.3% Adsorbent, E = Basal feed + Aflatoxin B1 (200 ppm) +0.4% Adsorbent, F = Basal feed + Aflatoxin B1 (200 ppm) +0.5% Adsorbent.

Clinical signs and behavior during aflatoxicoses has been investigated by many workers [2,12,13]. Increased water intake has also been reported in broiler chicks during aflatoxicosis [14]. A decrease in feed intake and increase in water intake may be due to avoid dehydration and replenish the water loss during watery and loose dropping Chohota *et al.* [15] described a general depression in broiler chicks suffering from aflatoxicosis. Robinson *et al.* [16] study demonstrated weakness, little response to feed and depression in wild birds suffering from aflatoxicosis. In our study group B having aflatoxin in feed exhibited abnormal signs and behavior throughout the course of experiment and was found sever at the end of 6^th^ week however groups D, E and F receiving aflatoxin and different levels of adsorbent in feed showed a very low cumulative score as compared to group B. The present study clearly indicated that the prepared adsorbent can play a vital role to ameliorate the ill effects of aflatoxin B1.

### Blood serum biochemical parameters

Serum biochemical parameters such as Total protein, Serum globulin, Albumen contents [17], alkaline phosphatase and alanine transferase [13] blood level can be used to determine the hepatic malfunctioning and liver injury. Adsorbents can effectively reduce the negative effects of aflatoxin in chicks consuming aflatoxin contaminated feed [13,18]. The effect of the prepared carbon nanostructures on different blood serum parameters are discussed below.

#### Alanine Transferase (ALT)

**Figure 1:**
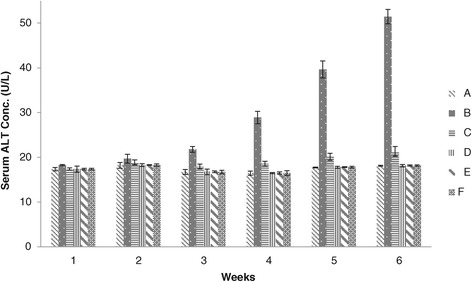
**Serum ALT concentration (U/L) in broiler chickens blood serum given different dietary levels of adsorbent prepared from bagasse.** (A = Basal feed, B = Basal feed + Aflatoxin B1 (200 ppm), C = Basal feed + Aflatoxin B1 (200 ppm) +0.2% Adsorbent, D = Basal feed + Aflatoxin B1 (200 ppm) +0.3% Adsorbent, E = Basal feed + Aflatoxin B1 (200 ppm) +0.4% Adsorbent, F = Basal feed + Aflatoxin B1 (200 ppm) +0.5% Adsorbent).

Weekly results of blood serum biochemistry analysis of broiler chicks given different levels of adsorbent revealed that the concentration of ALT of groups C, D, E, and F were significantly lower than group B throughout the course of experiment. The values of ALT concentration of groups C, D, E and F were noted non-significant at the end of 1^st^ and 2^nd^ weeks whereas a significant difference were observed at the end of 3^rd^, 4^th^, 5^th^ and 6^th^ weeks. ALT concentrations of groups D, E and F showed no significant difference, however lower values were observed in groups D and F (Figure 1).

Serum ALT and alkaline phosphatase activities, Total protein, serum albumens and globulin concentration have also been described as valuable parameters of liver injury and function [19]. The results of group B indicated high concentration of ALT fed on aflatoxin contaminated feed as compared to groups D, E and F offered feed containing aflatoxin and different levels of adsorbent. These results represented that addition of the prepared adsorbent in aflatoxin contaminated feed ameliorated the toxic effects of aflatoxin. Our findings are in close agreement to Pasha *et al.* [20], Gowda *et al.* [18], Ibrahim *et al.* [21], Shi *et al.* [22] and Watts *et al.* [23] who reported that different adsorbents have been found to diminish the toxic effects of aflatoxin in broiler chicks.

#### Alkaline Phosphatase (ALP)

**Figure 2:**
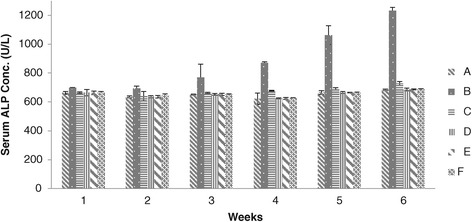
**Serum ALP concentration (U/L) in broiler chickens blood serum given different dietary levels of adsorbent prepared from bagasse.** (A = Basal feed, B = Basal feed + Aflatoxin B1 (200 ppm), C = Basal feed + Aflatoxin B1 (200 ppm) +0.2% Adsorbent, D = Basal feed + Aflatoxin B1 (200 ppm) +0.3% Adsorbent, E = Basal feed + Aflatoxin B1 (200 ppm) +0.4% Adsorbent, F = Basal feed + Aflatoxin B1 (200 ppm) +0.5% Adsorbent).

The results of ALP concentration has been shown in Figure 2. The ALP concentration of chicks of group B were significantly higher than that of groups C, D, E and F, while the values of ALP concentration of groups C, D, E and F were observed non-significant throughout the experimental weeks. Concentration of group A showed no significant difference to that of groups C, D, E and F. However lower values of ALP concentration were noted in groups D and E.

Biochemical changes in blood serum represent a stress on liver function [24,25]. In present study the activities of ALP increased, whereas the concentrations of total protein globulin and albumens in the serum were decreased in chicks of group B. All these are the prime indicators of aflatoxicosis [24,25] whereas increases ALP level are associated with the hepatobiliary malfunction. It has been also reported that though ALP is not a liver specific enzyme but always associated with liver injuries, biliary stasis and other nonspecific injuries to the body tissues [24]. Our results also demonstrated that chicks of groups D, E and F fed on diet containing aflatoxin and different levels of adsorbent diminished the toxic effects of aflatoxicosis. Same results have been reported by Miazzo *et al.* [7], Kubena *et al.* [12], Gowda *et al.* [18], Ibrahim *et al.* [21], Jindal *et al.* [24] and Abdel-Wahhab *et al.* [25] whose investigations showed that different adsorbent can effectively reduce the negative effects of aflatoxin in chicks consuming aflatoxin contaminated feed.

#### Serum albumen

**Figure 3:**
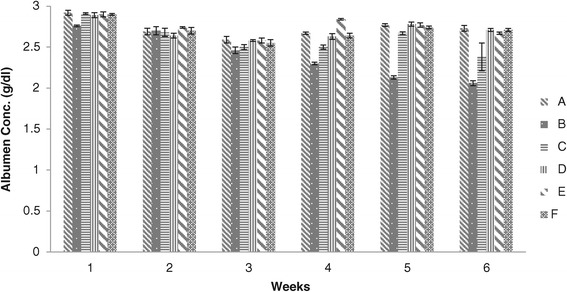
**Albumen concentration (g/dl) in broiler chickens blood serum given different dietary levels of adsorbent prepared from bagasse.** (A = Basal feed, B = Basal feed + Aflatoxin B1 (200 ppm), C = Basal feed + Aflatoxin B1 (200 ppm) +0.2% Adsorbent, D = Basal feed + Aflatoxin B1 (200 ppm) +0.3% Adsorbent, E = Basal feed + Aflatoxin B1 (200 ppm) +0.4% Adsorbent, F = Basal feed + Aflatoxin B1 (200 ppm) +0.5% Adsorbent).

The values of groups A, C, D, E and F were significantly higher than group B, while there no significant difference in the values of groups A, C, D, E and F at the end of 1^st^ week. At the end of 2^nd^ week no significant difference were seen in all groups however the values of groups B and C were found significantly less than that of groups A, D, E and F throughout the experimental period. The values of group C showed significant difference than that of group B during 1^st^, 3^rd^, 4^th^, 5^th^ and 6^th^ weeks (Figure 3).

Blood biochemical parameter alters in chicks consuming aflatoxin contaminated feed [18,24,26,27]. The decreased levels of serum total protein, globulin and albumen contents are the prime indicators of aflatoxicosis which can be reduced by the addition of adsorbent in aflatoxin contaminated feed [7,18,21,24,25,28]. Our findings showed that the level of albumen contents were found low in chicks of group B fed on diet containing aflatoxin alone whereas increased levels of albumen were noted in those groups offered feed containing aflatoxin along with different levels of adsorbent.

#### Serum total proteins

**Figure 4:**
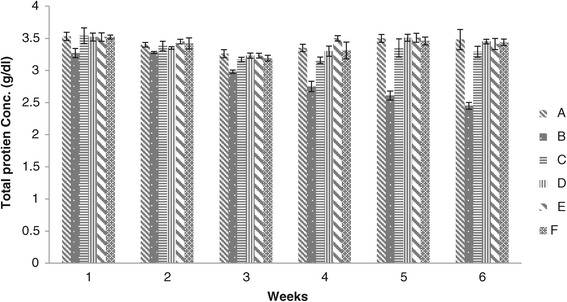
**Total protein concentration (g/dl) in broiler chickens blood serum given different dietary levels of adsorbent prepared from bagasse.** (A = Basal feed, B = Basal feed + Aflatoxin B1 (200 ppm), C = Basal feed + Aflatoxin B1 (200 ppm) +0.2% Adsorbent, D = Basal feed + Aflatoxin B1 (200 ppm) +0.3% Adsorbent, E = Basal feed + Aflatoxin B1 (200 ppm) +0.4% Adsorbent, F = Basal feed + Aflatoxin B1 (200 ppm) +0.5% Adsorbent).

Weekly results of Serum blood biochemistry of chicks for total protein concentration have been given in Figure 4. The results indicated that the total protein concentration of group B was significantly different than that of groups C, D, E and F, while concentration of total protein in blood serum of chicks of groups C, D, E and F were not significantly different at the end of 1^st^ week. Significant lower values were found in group C than that of groups D, E and F in the 5^th^ week. The concentration of group D was significantly less than that of groups E and F at the end of 2^nd^ week. Concentration of total protein of groups E and F were significantly different at the end of 4^th^ week, whereas the values of groups D, E and F showed no significant difference at the end of 6^th^ week. High values of total protein concentration were observed in groups D and E. The levels of serum albumin and total protein are the sensitive indicators of aflatoxicosis. In the present study the level of serum total protein decreased in group B consuming aflatoxin contaminated feed when compared to Groups C, D, E, and F fed on diet containing aflatoxin and different levels of adsorbent.

Decrease concentration of serum total protein has been proposed as indicator of inhibition of protein synthesis [18]. Our results showed that addition of adsorbent to aflatoxin contaminated feed strongly ameliorated the hazardous effect of aflatoxin. Various reports are available which demonstrated that different adsorbents have the capability to reduce the toxic effect in chicks when offered aflatoxin contaminated feed [7,18,20].

#### Serum globulin

**Figure 5:**
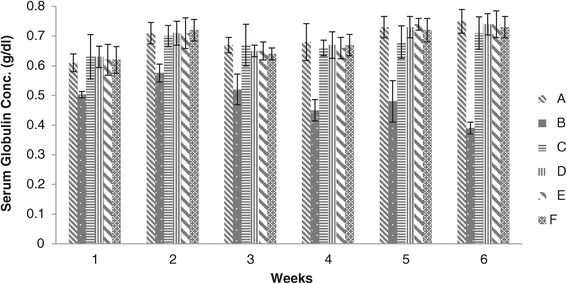
**Serum Globulins concentration (g/dl) in broiler chickens blood serum given different dietary levels of adsorbent prepared from bagasse.** (A = Basal feed, B = Basal feed + Aflatoxin B1 (200 ppm), C = Basal feed + Aflatoxin B1 (200 ppm) +0.2% Adsorbent, D = Basal feed + Aflatoxin B1 (200 ppm) +0.3% Adsorbent, E = Basal feed + Aflatoxin B1 (200 ppm) +0.4% Adsorbent, F = Basal feed + Aflatoxin B1 (200 ppm) +0.5% Adsorbent).

Weekly Serum globulins result of chicks given different levels of adsorbent has been presented in Figure 5. Serum globulins concentration in blood serum of chicks of group B were found significantly less than that of groups C, D, E and F. The concentration of serum globulins in blood serum of broiler chicken of groups C, D, E and F showed no significant difference throughout the course of study however the highest values for serum globulins were determined in groups D and E. Our results showed that the serum globulin levels decreased in group B fed on aflatoxin contaminated feed, while increased in groups C, D, E and F by the addition of the prepared adsorbent.

According to Yinghua *et al.* [28] the levels of serum globulin decreases when chick fed diets containing aflatoxin whereas no significant difference were seen between control and that of chicks fed on diets containing aflatoxin plus adsorbent.

### Mortality

**Figure 6:**
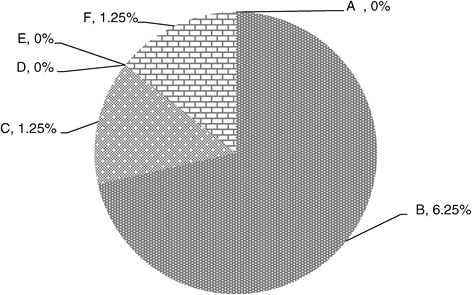
**Relative % mortality of broiler chickens given different levels of adsorbent prepared from bagasse.** (A = Basal feed, B = Basal feed + Aflatoxin B1 (200 ppm), C = Basal feed + Aflatoxin B1 (200 ppm) +0.2% Adsorbent, D = Basal feed + Aflatoxin B1 (200 ppm) +0.3% Adsorbent, E = Basal feed + Aflatoxin B1 (200 ppm) +0.4% Adsorbent, F = Basal feed + Aflatoxin B1 (200 ppm) +0.5% Adsorbent).

Weekly results of mortalities of broiler chickens at different level of adsorbent have been presented in Figure 6. There was no mortality in the groups A, C, D, E and F throughout the experimental period. Total mortality of broiler chickens in group B was 2 at the end of 6^th^ week which was recorded as 3.07%. These results are in accordance with the findings of Afzal *et al.* [29] according to which percent mortality increases in chicks fed on aflatoxin contaminated feed whereas percent mortality can be decreased when fed on diet containing aflatoxin plus adsorbents.

Mortality in chicks fed on aflatoxin contaminated feed has been also reported by many workers [29,30]. Chicks of group B consuming aflatoxin contaminated feed without adsorbent showed high percent mortality as compared to groups C, D, E and F fed with aflatoxin plus different levels of adsorbent.

### Body weights

**Figure 7:**
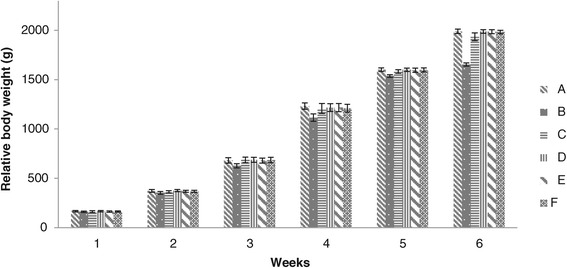
**Relative body weight of broiler chickens given different levels of adsorbent prepared from bagasse.** (A = Basal feed, B = Basal feed + Aflatoxin B1 (200 ppm), C = Basal feed + Aflatoxin B1 (200 ppm) +0.2% Adsorbent, D = Basal feed + Aflatoxin B1 (200 ppm) +0.3% Adsorbent, E = Basal feed + Aflatoxin B1 (200 ppm) +0.4% Adsorbent, F = Basal feed + Aflatoxin B1 (200 ppm) +0.5% Adsorbent).

Relative body weight of group B showed significant lesser body weight fed on toxin contaminated feed through the experimental period. Group D values were significantly higher than that of groups B, C, E and F fed on aflatoxin and different levels of adsorbent at the end of 2^nd^ week. During 3^rd^ week all the values of all groups were found non-significant except group B. The body weight of broiler chicks of groups D, E and F showed no significant difference, while the values of all these groups were significantly higher than that of groups B and C during 5^th^ week. No significant differences were noted in groups A and D, E and F whereas values of these groups were found significant higher than that of groups B and C at the end of 6^th^ week (Figure 7).

The body weight of chicks decreased when fed on aflatoxin contaminated feed as previously reported by many workers [7,26,27,31]. Our study showed that body weight of chicks of group B decreased throughout the course of study whereas those groups receiving aflatoxin contaminated feed along with different levels of adsorbent showed increase in body weight. Aflatoxins are prim concern to poultry industry because of their negative effects. The lower weight gains in chicks fed with aflatoxin alone as compared to other groups were consistent with previous studies on the toxic effects of aflatoxin [26,27,31]. They also investigated a trend in improvement in body weight gain when fed with adsorbent added to aflatoxin contaminated feed.

### Liver weight

**Figure 8:**
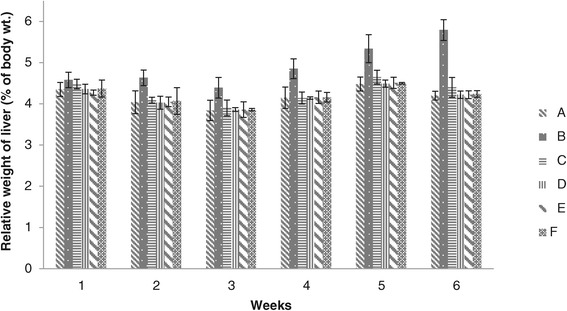
**Relative weight of liver (% body weight) of broiler chicks given different levels of adsorbent prepared from bagasse.** (A = Basal feed, B = Basal feed + Aflatoxin B1 (200 ppm), C = Basal feed + Aflatoxin B1 (200 ppm) +0.2% Adsorbent, D = Basal feed + Aflatoxin B1 (200 ppm) +0.3% Adsorbent, E = Basal feed + Aflatoxin B1 (200 ppm) +0.4% Adsorbent, F = Basal feed + Aflatoxin B1 (200 ppm) +0.5% Adsorbent).

Figure 8 data revealed that weekly results of relative weight of liver of chicks given different levels of adsorbent were non-significant at the end of 1^st^ week among all groups (A, B, C, D, E and F). Significantly higher values were recorded in group B at the end of 2^nd^ week and continued till the end of 6^th^ week. The values of groups C, D, E and F were found non-significant, while low values of relative liver weight were determined in groups D and E. Liver is the main target organ of aflatoxin. In addition, the relative weight of liver in poultry has been reported to be increased by the presence of aflatoxin in the feeding diet.

Relative weight of liver in groups D, E and F fed on diet containing aflatoxin plus different levels of adsorbent showed no pathological signs which confirmed that addition of adsorbent to aflatoxin contaminated feed diminish the toxic effects of aflatoxin [32,33]. The basic theory is that adsorbent detoxify aflatoxin contaminated feed by binding them strongly enough to prevent toxic interaction with the consuming animals and to prevent aflatoxin absorption from the gastrointestinal tract [32].

### Kidney weight

**Figure 9:**
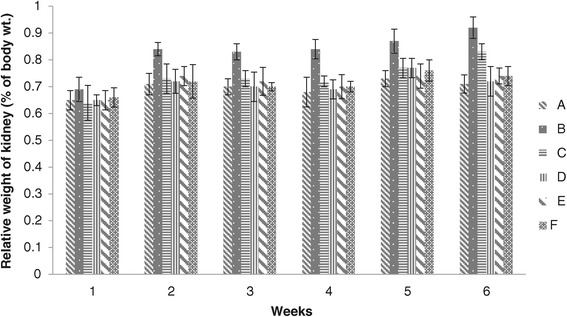
**Relative weight of kidney (% body weight) of broiler chickens given different levels of adsorbent prepared from bagasse.** (A = Basal feed, B = Basal feed + Aflatoxin B1 (200 ppm), C = Basal feed + Aflatoxin B1 (200 ppm) +0.2% Adsorbent, D = Basal feed + Aflatoxin B1 (200 ppm) +0.3% Adsorbent, E = Basal feed + Aflatoxin B1 (200 ppm) +0.4% Adsorbent, F = Basal feed + Aflatoxin B1 (200 ppm) +0.5% Adsorbent).

The results of relative weight of kidney showed that values of all groups were non-significant at the end of 1^st^ week, while the values of group B were found significantly higher than other groups at the end of 2^nd^ to 6^th^ weeks. No significant difference were seen among groups A, C, D, E and F at the end of 5^th^ week, whereas significant difference was noted between group B and groups A, D, E and F at the end of 6^th^ week. Groups D, E and F values were found non-significant, however low values were recorded in groups D and E. The relative weights of kidney of group B were significantly increased in chicks fed on aflatoxin B1 alone (Figure 9).

The results abstained were in agreements with previous study reported by Huff *et al.* [34] in which he found that aflatoxin alone in feed increase the kidney weight whereas by addition of adsorbent to aflatoxin contaminated feed ameliorated the deleterious effects of aflatoxin in broiler chicks. The increased relative weight of kidney was probably due to slight renal damage [34].

### Spleen weight

**Figure 10:**
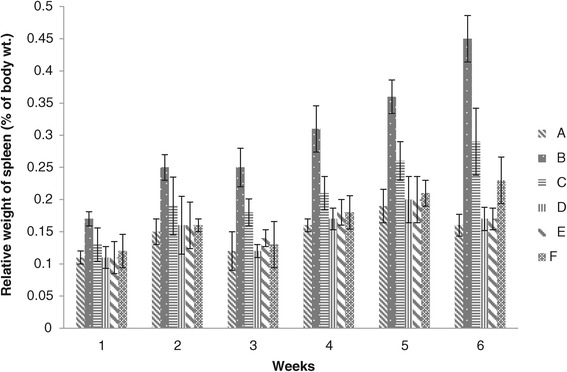
**Relative weight of spleen (% body weight) of broiler chickens given different levels of adsorbent prepared from bagasse.** (A = Basal feed, B = Basal feed + Aflatoxin B1 (200 ppm), C = Basal feed + Aflatoxin B1 (200 ppm) +0.2% Adsorbent, D = Basal feed + Aflatoxin B1 (200 ppm) +0.3% Adsorbent, E = Basal feed + Aflatoxin B1 (200 ppm) +0.4% Adsorbent, F = Basal feed + Aflatoxin B1 (200 ppm) +0.5% Adsorbent).

The results of relative weight of spleen of groups A and B were found significantly different, values of group B were noted significantly higher than that of group A and were recorded maximum at the end of 6^th^ week. Group C values were non-significant to group A, D, E, and F at the end of 2^nd^ week, whereas a significant higher values were noted at the end of 3^rd^, 4^th^, 5^th^ and 6^th^ weeks. The results also revealed that values of groups D, E and F were not significantly different, however lower values were recorded in groups D and E (Figure 10).

Our findings related to group B are in close agreement to Hesham *et al.* [27] in which the increase in relative spleen weight was due to the presences of aflatoxin in the diet. The results of groups D, E and F showed that addition of the prepared adsorbent in diet ameliorated the toxic effects of aflatoxin B1. These results were in accordance with the work of Miazzo *et al.* [7], Pasha *et al.* [20] in which they studied that adsorbents are effective in preventing or ameliorating the changes in organs weights of chicks fed on aflatoxin B1 contaminated feed.

### Weight of Bursa of fabricious

**Figure 11:**
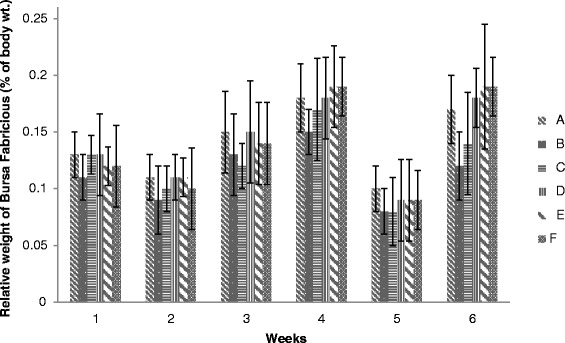
**Relative weight of Bursa fabricious (% body weight) of broiler chickens given different levels of adsorbent prepared from bagasse.**

Results of relative weight of Bursa of fabricious have been shown in Figure 11. The values of Bursa of fabricious weight of broiler chicks given different levels of adsorbent were non-significant among all the groups (A, B, C, D, E and F). No significant change were found throughout the course of study, however the values of groups D and E were closely resembled to that of group A. These results are in close agreement to Bailey *et al.* [35] in which they investigated that the relative weights of Bursa were not affected by any dietary treatments.

### Hemorrhages in organs

Results of hemorrhages in organs have been presented in Table 3. Different organs including liver, Kidney and heart of chicks of groups A and E exhibited normal gross morphology throughout the experimental weeks. Hemorrhages on liver of chicks of group B started during 2^nd^ week, while that of kidney in the 3^rd^ week. The intensity was found increased during 4^th^ and 5^th^ week and recorded maximum at the end of 6^th^ week. However the heart was remained normal throughout the course of experiment. Hemorrhages on liver of chicks of group C started during 3^rd^ week, while that on kidney in the 5^th^ week and persisted till the end of 6^th^ week. However the intensity was very low as compared to that of group B. The results of groups D, E and F were noted very close to each other, whereas group D presented close values to that of group A.

**Table 3:** **Hemorrhages in organs of broiler chickens given different level of adsorbent prepared from bagasse**

**Time period**	**Organs**	**Maximum possible score**	**Groups**					
			**A**	**Aflatoxin B1 (AFB1) 200 μg/kg**				
**Week(Days)**								
				**B**	**C**	**D**	**E**	**F**
1(7)	Liver	9	0	0	0	0	0	0
	Kidney	9	0	0	0	0	0	0
	Heart	9	0	0	0	0	0	0
**Cumulative score**		**27**	**0**	**0**	**0**	**0**	**0**	**0**
2(14)	Liver	9	0	2	0	0	0	0
	Kidney	9	0	0	0	0	0	0
	Heart	9	0	0	0	0	0	0
**Cumulative score**		**27**	**0**	**2**	**0**	**0**	**0**	**0**
3(21)	Liver	9	0	3	1	0	0	0
	Kidney	9	0	2	0	0	0	0
	Heart	9	0	0	0	0	0	0
**Cumulative Score**		**27**	**0**	**5**	**1**	**0**	**0**	**0**
4(28)	Liver	9	0	7	2	0	0	0
	Kidney	9	0	3	0	0	0	0
	Heart	9	0	0	0	0	0	0
**Cumulative score**		**27**	**0**	**10**	**2**	**0**	**0**	**0**
5(35)	Liver	9	0	9	2	0	0	1
	Kidney	9	0	4	0	0	0	0
	Heart	9	0	0	0	0	0	0
**Cumulative score**		**27**	**0**	**13**	**2**	**0**	**0**	**1**
6(42)	Liver	9	0	9	4	3	1	2
	Kidney	9	0	5	1	0	0	0
	Heart	9	0	0	0	0	0	0
**Cumulative score**		**27**	**0**	**14**	**5**	**3**	**1**	**2**

(A = Basal feed, B = Basal feed + Aflatoxin B1 (200 ppm), C = Basal feed + Aflatoxin B1 (200 ppm) +0.2% Adsorbent, D = Basal feed + Aflatoxin B1 (200 ppm) +0.3% Adsorbent, E = Basal feed + Aflatoxin B1 (200 ppm) +0.4% Adsorbent, F = Basal feed + Aflatoxin B1 (200 ppm) +0.5% Adsorbent).

Results showed that tendency of hemorrhages on different organs except heart of group B chicks were found maximum, while that of groups D, E and F consuming aflatoxin and different levels of adsorbent in feed remained normal as compared to group B score. Hemorrhages on different organs in broiler chicks during aflatoxicosis have also been reported by Ortatatli and Oguz [2]. Impairment of coagulation mechanism and hematopoieses increased indothelial cells fragility which might be responsible for tendency of hemorrhages in the tissues [2].

### Enlargement of organs

Different organs of chicks of groups A, E and F exhibited normal size and appearance throughout the course of experimental period. Gross examination of chicks liver of group B showed slight enlargement from the 1^st^ week, whereas that of kidney from the 3^rd^ week. The intensity of enlargement of liver and kidney of group B increased after 4^th^ and 5^th^ weeks and found maximum at the end of 6^th^ week as compared to that of groups A, C, D, E and F. The size and appearance of chicks’ heart of all groups (A, B, C, D, E and F) were found normal which persisted throughout experimental weeks (Table 4).

**Table 4:** **Hemorrhages in organs of broiler chickens given different level of adsorbent prepared from bagasse**

**Time period**	**Organs**	**Maximum possible score**	**Groups**					
			**A**	**Aflatoxin B1 (AFB1) 200 μg/kg**				
**Week (Days)**								
				**B**	**C**	**D**	**E**	**F**
1(7)	Liver	9	0	0	0	0	0	0
	Kidney	9	0	0	0	0	0	0
	Heart	9	0	0	0	0	0	0
**Cumulative score**		**27**	**0**	**0**	**0**	**0**	**0**	**0**
2(14)	Liver	9	0	2	0	0	0	0
	Kidney	9	0	0	0	0	0	0
	Heart	9	0	0	0	0	0	0
**Cumulative score**		**27**	**0**	**2**	**0**	**0**	**0**	**0**
3(21)	Liver	9	0	3	1	0	0	0
	Kidney	9	0	2	0	0	0	0
	Heart	9	0	0	0	0	0	0
**Cumulative Score**		**27**	**0**	**5**	**1**	**0**	**0**	**0**
4(28)	Liver	9	0	7	2	0	0	0
	Kidney	9	0	3	0	0	0	0
	Heart	9	0	0	0	0	0	0
**Cumulative score**		**27**	**0**	**10**	**2**	**0**	**0**	**0**
5(35)	Liver	9	0	9	2	0	0	1
	Kidney	9	0	4	0	0	0	0
	Heart	9	0	0	0	0	0	0
**Cumulative score**		**27**	**0**	**13**	**2**	**0**	**0**	**1**
6(42)	Liver	9	0	9	4	3	1	2
	Kidney	9	0	5	1	0	0	0
	Heart	9	0	0	0	0	0	0
**Cumulative score**		**27**	**0**	**14**	**5**	**3**	**1**	**2**

(A = Basal feed, B = Basal feed + Aflatoxin B1 (200 ppm), C = Basal feed + Aflatoxin B1 (200 ppm) +0.2% Adsorbent, D = Basal feed + Aflatoxin B1 (200 ppm) +0.3% Adsorbent, E = Basal feed + Aflatoxin B1 (200 ppm) +0.4% Adsorbent, F = Basal feed + Aflatoxin B1 (200 ppm) +0.5% Adsorbent).

(A = Basal feed, B = Basal feed + Aflatoxin B1 (200 ppm), C = Basal feed + Aflatoxin B1 (200 ppm) +0.2% Adsorbent, D = Basal feed + Aflatoxin B1 (200 ppm) +0.3% Adsorbent, E = Basal feed + Aflatoxin B1 (200 ppm) +0.4% Adsorbent, F = Basal feed + Aflatoxin B1 (200 ppm) +0.5% Adsorbent).

Liver enlargements of intoxicated broiler chicks followed by moderate kidney enlargement during aflatoxicosis have also been documented by Ortatatli and Oguz [2] Enlarged kidney and liver in group B were also evident by the significant increase in relative organs weight of chicks of group B consuming aflatoxin contaminated feed. Enlarged livers and kidneys due to aflatoxicosis have also been reported in other birds like Japanese quil, ducks and water fowls [2,12,36]. Our results also demonstrated that by adding different levels of adsorbent in aflatoxin contaminated feed ameliorated the hazardous effects of aflatoxin as previously, reported by [12,36] in which they studied the ameliorating effects of different adsorbents in checks fed on aflatoxin contaminated feed.

## Conclusions

In this study carbon nanocomposites were fed to poultry birds along with aflatoxin B1 and different physiological and blood parameters were monitored. The results of broiler chicks of group D fed on 0.3% of the prepared adsorbent/kg feed were highly significant to group B fed on aflatoxin B1 (200 μg/Kg feed) contaminated feed. Whereas the data of group A (fed on normal basal diet) showed no significant difference to that of group D. The results clearly indicated that the prepared adsorbent 0.3%/ kg feed is highly effective to adsorb and detoxify aflatoxin B1 in gastrointestinal tract of broiler chickens and pass safely leaving no harmful effects. However the results of groups E and F fed on 0.4% and 0.5% respectively showed slight variation in tested parameters from group A. From the results it is concluded that the prepared adsorbent can be used as alternative of powdered activated carbon for the detoxification of aflatoxin in poultry feed as the former cause dehydration and salt deficiencies when administered to poultry birds.

## Methods

### Preparation of adsorbent

Bagasse was used as biomass to produce magnetic carbon nanocomposite. The biomass was shredded and transferred to ethanolic FeCl_3_.6H_2_O (10% w/v). After 30 minutes biomass was separated and air dried at room temperature for 24 hours. Thereafter, biomass was charred in a specially designed chamber (designed by the author) consisting of a stainless steel container equipped with an electric heater, wire gauze, nitrogen gas (to provide a nitrogen rich atmosphere) inlet and exhaust outlet. The prepared adsorbent was characterized by surface area analyzer, SEM, XRD, FTIR, TG/DTA and EDX.

### Production of aflatoxin B1

Aflatoxin B1 was produced from *A. flavous* by culturing on rice following the method of Shot Well [2]. The Aflatoxin B1 residues produced were suspended into poly ethylene glycol and the required quantities were evenly mixed with basal feeds to get different level of aflatoxin B1contaminated feed samples. The preparation of basal feed without toxin binder were ordered and purchased from feed mill and checked for Aflatoxin B1 ≤ 1 μg/kg.

### Induction of aflatoxicoses in broiler checks

All animal experiments were carried out according to the Scientific Procedures Issue-1 of Animal Bylaws-2008 approved by the legal bodies of the University of Malakand Khyber Pakhtoonkhwa, Pakistan. The ethical committee of the department of Biochemistry granted approval for conducting this study under the said protocols (ProceduresIssue-1 of Animal Bylaws-2008). A total of 480 one day old broiler chicks were purchased from local hatchery (Rawalpendi, Pakistan) and were kept in poultry house under standard environmental conditions. All these chicks were then equally divided into six different groups (A-F) and given access to fresh water *ad libitum.*

### Experimental protocol

Broiler chickens of different groups were given the following experimental diets.

- **Group A:** Chicks were kept on normal feed throughout study duration.
- **Group B:** Chicks were kept on Aflatoxin B1 (200 μg/kg feed) contaminated feed for 6 weeks.
- **Group C:** Chicks were given Aflatoxin B1 (200 μg/kg feed) contaminated feed mixed with 0.2% of the prepared adsorbent throughout study duration.
- **Group D:** Chicks were given Aflatoxin B1 (200 μg/kg feed) contaminated feed mixed with 0.3% of the prepared adsorbent throughout 6 weeks.
- **Group E:** Chicks were given Aflatoxin B1 (200 μg/kg feed) contaminated feed mixed with 0.4% of the prepared adsorbent throughout study duration.
- **Group F:** Chicks were given Aflatoxin B1 (200 μg/kg feed) contaminated feed mixed with 0.5% of the prepared adsorbent throughout study period.

### Observations and analysis

The body weights and mortality of the checks were recorded at the end of every week, while blood serum biochemistry for alanine transferase, alkaline phosphatase, albumen, globulin and total protein were carried out by collecting blood of 3 chicks individually from each group at the end of 1^st^ to 6^th^ weeks using commercially available kits (Diasys Diagnostic system Germany) [37].

#### Clinical signs

Clinical signs and behavior of chicks given different levels of adsorbent (alertness, Attraction to feed and water, faeces consistency and feather condition) were evaluated by scoring 0 to 3 based upon (0) absence, (1) mild, (2) moderate and (3) maximum. All these observations were carried out at the end of each experimental week [14,38].

#### Necropsy of chicks

At the end of experimental weeks three chicks were individually weighed and sacrificed from each group by cervical dislocation. Different organs (liver, Spleen, Kidney and Bursa of Fabricious) were harvested and weighed to determined relative weight. The liver, kidney and heart were collected and scored for gross lesion as described in clinical signs [14,38].

#### Statistical analysis

The data were analyzed for descriptive statistics, analysis of variance tests and LSD All-pair wise comparisons test using STATISTIX 8.1 package. The data were considered significantly different at P-value ≤0.05. The scores for clinical signs and gross lesions were compared with control group on arithmetical difference bases.
